# Supplementary material for: Orbital disproportionation of electronic density is a universal feature of alkali-doped fullerides
Source: Nat Commun. 2016 Oct 7;7:13093. doi: 10.1038/ncomms13093 (PMC5059769; doi:10.1038/ncomms13093)
Supplement: Supplementary Information — Supplementary Figures 1-7, Supplementary Tables 1-2, Supplementary Notes 1-2, Supplementary Methods and Supplementary References [file ncomms13093-s1.pdf]

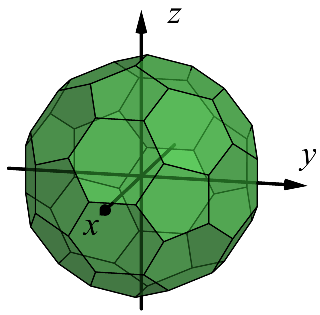

Supplementary Figure 1. Coordinate system of C_{60}.

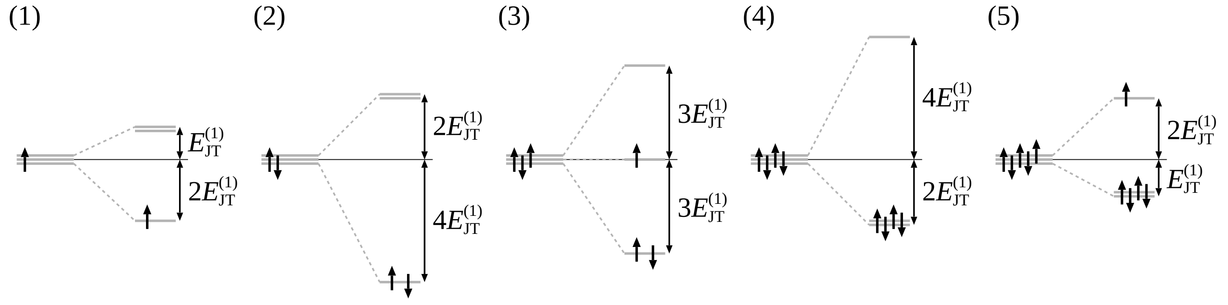

Supplementary Figure 2. JT splitting of C_{60}^{n-}. $E_{JT}^{(1)} = 50.2$ meV is the JT stabilization energy of C_{60}^{-}.

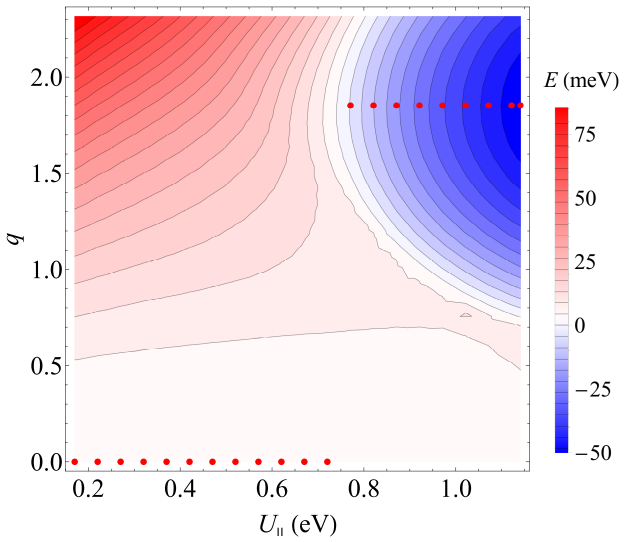

Supplementary Figure 3. Ground state energy of K_3C_{60}. The total energy E_g of fcc K_3C_{60} is plotted as a function of the Jahn-Teller deformation q and $U_\parallel$ (in meV). For each $U_\parallel$, the energy E_g at $q = 0$ is subtracted from E_g. The red points show the ground state for each $U_\parallel$. For small $U_\parallel$, the JT distortion is suppressed, whereas for $U_\parallel \gtrsim 0.75$ eV, the JT distortion is favored. Therefore, in the former region of $U_\parallel$, the orbitals are degenerate and equally populated in the ground state, while in the latter region, the LUMO levels are completely split and orbital disproportionation arises.

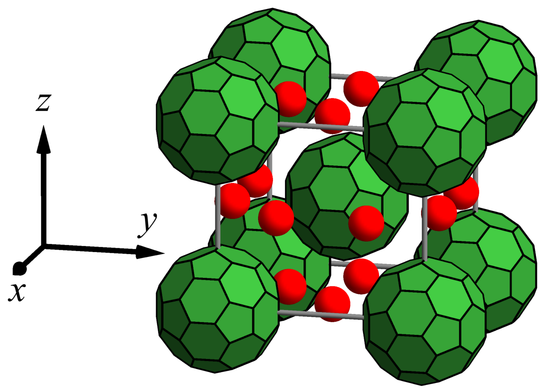

Supplementary Figure 4. Structure of bct K_4C_{60}. The green balls are fullerene C_{60} and the red spheres are K atoms which locate at $a(1/2, 0.218152, 0)$ and equivalent positions with respect to the central C_{60}.

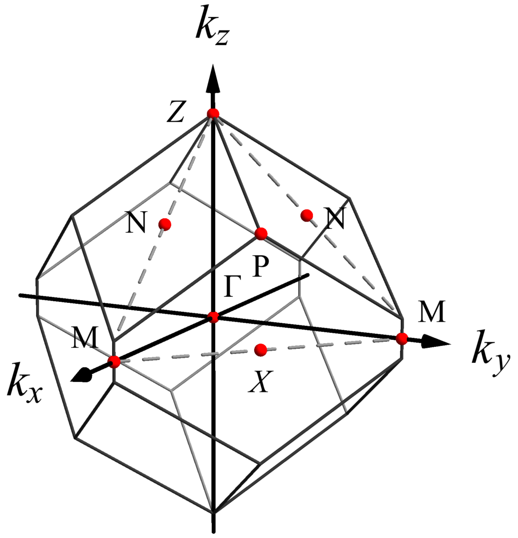

Supplementary Figure 5. The first Brillouin zone of bct lattice. The band is plotted along the path $Z = (0, 0, (1 + (a/c)^2)c/(2a)) \to \Gamma = (0, 0, 0) \to M = (1, 0, 0) \to P = (1/2, 1/2, c/(2a)) \to M = (0, 1, 0) \to \Gamma \to X = (1/2, 1/2, 0) \to P \to N = (1/2, 0, c/(2a)) \to Z \to N = (0, 1/2, c/(2a)) \to P \to Z$ in the unit of $2\pi/a$.

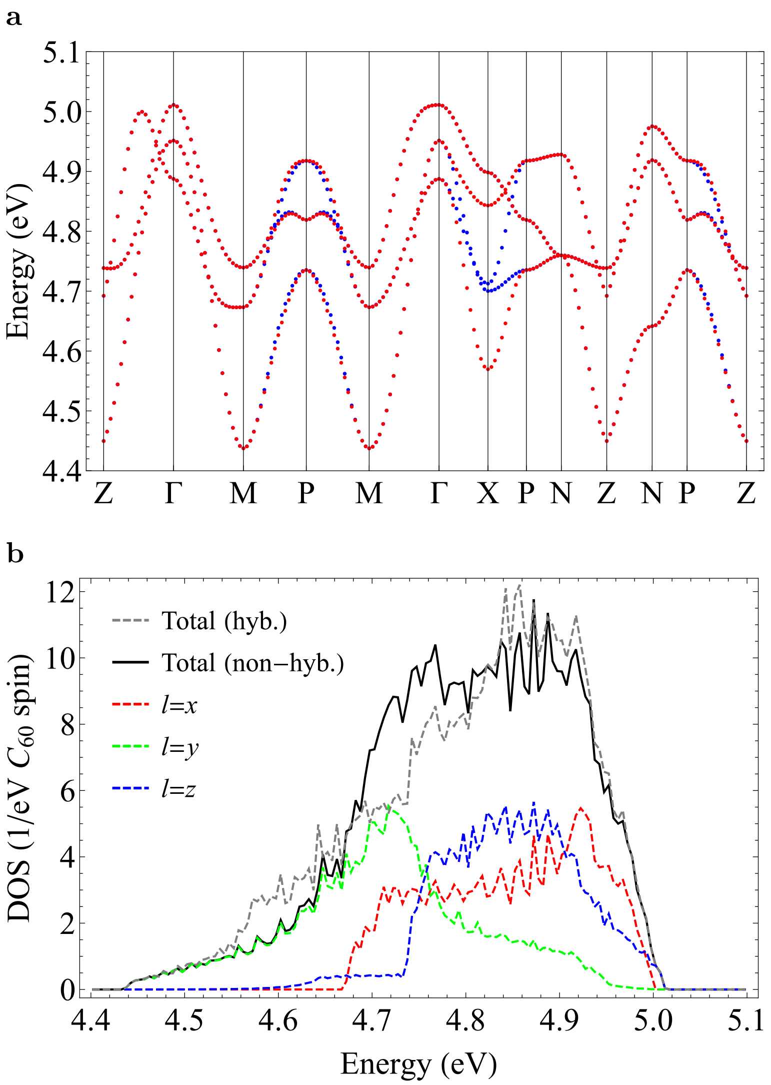

Supplementary Figure 6. The effect of the interorbital hybridization on the LUMO band. (**a**) The band structures and (**b**) the density of states (DOS) of bct K_4C_{60} with and without hybridization. The Jahn-Teller splitting is not taken into account. (**a**) The red and blue dots indicate the presence and the absence of the hybridization between the x orbital and the other orbitals, respectively. (**b**) The gray dashed line is the total DOS with hybridization, the black solid line is the total DOS without hybridization, the red, green, and blue dashed lines indicate the partial DOS.

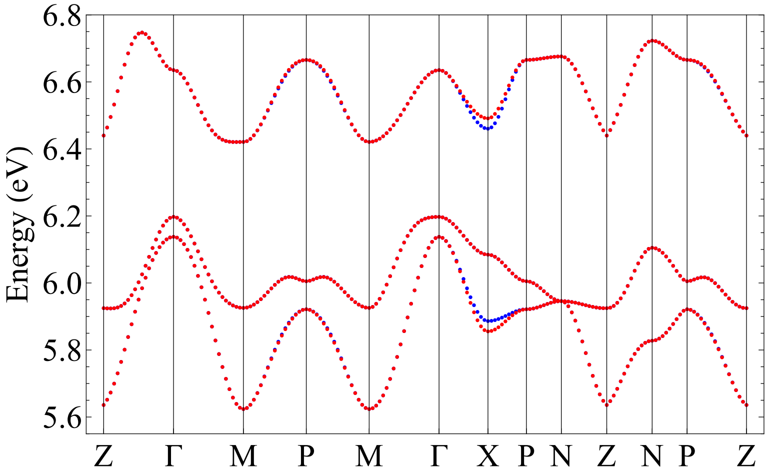

Supplementary Figure 7. The effect of the hybridization on the split LUMO band. The band structure with the splitting of the orbital levels. The hybridized (red) and non-hybridized (blue) band structures are similar to each other.

Supplementary Table 1. Transfer parameters of bct K_4C_{60}. The LUMO levels (eV) and transfer parameters (meV) of bct K_4C_{60} derived by fitting the DFT calculations into model Hamiltonian.

ϵ_x	ϵ_y	ϵ_z	t_{xx}	t_{yy}	t_{zz}	t_{xy}	t_{yz}	t_{zx}
4.849	4.715	4.847	13.4	32.1	17.0	−17.1	14.6	0.0
t'^x_{xx}	t'^x_{yy}	t'^x_{zz}	t'^y_{xx}	t'^y_{yy}	t'^y_{zz}	t'^z_{xx}	t'^z_{yy}	t'^z_{zz}
14.4	7.5	−9.0	−2.6	6.1	14.8	51.3	8.8	19.8

Supplementary Table 2. Static Jahn-Teller effect of C_{60}^{n-} anions. Jahn-Teller distortion in polar and Cartesian coordinates, occupation numbers, and JT stabilization energies (meV) of C_{60}^{n-}. For the calculations of the Cartesian coordinates, we set the three Euler angles γ, θ, ϕ zero, leading to $q_\xi = q_\eta = q_\zeta = 0$.

n	(q, α)	(q_θ, q_ϵ)	(n_1, n_2, n_3)	$E_{\rm JT}$
1	$(g, 0)$	$(g, 0)$	(0,0,1)	$E_{\rm JT}^{(1)}$
2	$(2g, 0)$	$(2g, 0)$	(0,0,2)	$4E_{\rm JT}^{(1)}$
3	$(\sqrt{3}g, \pi/2)$	$(0, \sqrt{3}g)$	(2,0,1)	$3E_{\rm JT}^{(1)}$
4	$(2g, \pi)$	$(-2g, 0)$	(2,2,0)	$4E_{\rm JT}^{(1)}$
5	(g, π)	$(-g, 0)$	(2,2,1)	$E_{\rm JT}^{(1)}$

SUPPLEMENTARY NOTE 1

Jahn-Teller effect of isolated C_{60}^{n-}

Isolated C_{60} molecule (I_h symmetry) has triply degenerate t_{1u} LUMO level. The t_{1u} orbital couples to two nondegenerate a_g normal vibrational modes and eight five-fold degenerate h_g normal vibrational modes. In this work, we omit the a_g modes and use effective h_g mode. The Jahn-Teller (JT) Hamiltonian of C_{60}^{n-} ($n = 1-5$) is written as

$$H_{\mathrm{JT}} = \sum_\gamma \frac{\hbar\omega}{2}\left(p_\gamma^2 + q_\gamma^2\right) + \hbar\omega g \sum_\sigma \left(\hat{c}_{x\sigma}^\dagger, \hat{c}_{y\sigma}^\dagger, \hat{c}_{z\sigma}^\dagger\right) \begin{pmatrix} \frac{1}{2}q_\theta - \frac{\sqrt{3}}{2}q_\epsilon & -\frac{\sqrt{3}}{2}q_\zeta & -\frac{\sqrt{3}}{2}q_\eta \\ -\frac{\sqrt{3}}{2}q_\zeta & \frac{1}{2}q_\theta + \frac{\sqrt{3}}{2}q_\epsilon & -\frac{\sqrt{3}}{2}q_\xi \\ -\frac{\sqrt{3}}{2}q_\eta & -\frac{\sqrt{3}}{2}q_\xi & -q_\theta \end{pmatrix} \begin{pmatrix} \hat{c}_{x\sigma} \\ \hat{c}_{y\sigma} \\ \hat{c}_{z\sigma} \end{pmatrix}, \tag{1}$$

where ω is the frequency, g is the dimensionless vibronic coupling constant, $(q_\theta, q_\epsilon, q_\xi, q_\eta, q_\zeta)$ are the dimensionless mass-weighted normal vibrational coordinates which transform as $2z^2 - x^2 - y^2$, $x^2 - y^2$, yz, zx, xy, respectively, under symmetric operations (for the definition of the coordinates see Supplementary Figure 1) and p_γ ($\gamma = \theta, \epsilon, \xi, \eta, \zeta$) is the conjugate momentum of q_γ. The components $\theta, \epsilon, \xi, \eta, \zeta$ of h_g mode are denoted 1,4,5,2,3, respectively, in Ref. 1.

The h_g normal coordinates can be transformed into polar coordinates $(q, \alpha, \gamma, \theta, \phi)$.[1] Under appropriate rotation of the electronic coordinates $\lambda = x, y, z$,

$$\hat{c}_{l\sigma}^\dagger = \sum_{\lambda'=x,y,z} S_{l\lambda}(\gamma, \theta, \phi)\hat{c}_{\lambda\sigma}^\dagger, \tag{2}$$

with

$$S_{l\lambda}(\gamma, \theta, \phi) = \left[B_P(\gamma)C_P(\theta)D_P(\phi)\right]_{l\lambda}, \tag{3}$$

we obtain adiabatic electronic states $l = 1, 2, 3$. Here, the rotation matrices B_P, C_P, D_P are the same as in Ref. 1 (angle θ and the component θ of h_g coordinate are different from each other). By the unitary transformation

$$\tilde{H}_{\mathrm{JT}} = \hat{S}^\dagger \hat{H}_{\mathrm{JT}} \hat{S}, \tag{4}$$

the potential term of the JT Hamiltonian (1) becomes diagonal:

$$U_{\mathrm{JT}} = \frac{\hbar\omega}{2}q^2 + \hbar\omega g q \sum_\sigma \left[\cos\left(\alpha + \frac{\pi}{3}\right)\hat{n}_{1\sigma} + \cos\left(\alpha - \frac{\pi}{3}\right)\hat{n}_{2\sigma} - \cos\alpha\hat{n}_{3\sigma}\right]. \tag{5}$$

The range of α is $0 \geq \alpha < \pi/3$ or equivalent range in the configuration space.

Minimizing Eq. (5) under the condition of $2(n_1 + n_2 + n_3) = n$, the JT deformation and the JT stabilization energy are obtained as follows.[2] Here, n_l is an occupation number of electron ($n_l = 0, 1$). For example, when there is one electrons in the LUMO orbitals (C_{60}^-), the amplitude of the JT coordinates at the minima of the adiabatic potential energy surface (5) is

$$(q, \alpha) = (g, 0), \tag{6}$$

with the occupation numbers

$$(n_1, n_2, n_3) = (0, 0, 1). \tag{7}$$

The JT stabilization energy (the gain by the deformation) is

$$E_{\mathrm{JT}} = E_{\mathrm{JT}}^{(1)} = \frac{\hbar\omega g^2}{2}. \tag{8}$$

In the case of C_{60} anion, the effective $g = 1.07$ and $\omega = 87.7$ meV, the stabilization energy $E_{\mathrm{JT}}^{(1)} = 50.2$ meV.

By the same procedure, we obtain the JT deformations, occupations, and JT stabilization energies for all cases (Supplementary Table 2).

In the strong JT coupling limit ($g \to \infty$), the ground state is well described in the space of the ground adiabatic state. Within the approximation, the kinetic term of Eq. (4) can be separated into radial and rotational parts in the configuration space of the h_g mode.[1] In the case of $n = 1, 2, 4, 5$, there are three dimensional radial part and two dimensional rotational part (Eqs. (12) and (24) in Ref. 1): for $n = 1, 5$,

$$\begin{aligned} \tilde{H}_{\mathrm{KE}} = &-\frac{\hbar\omega}{2}\left[\frac{1}{q^2}\frac{\partial}{\partial q}\left(q^2\frac{\partial}{\partial q}\right) + \frac{1}{q^2 \sin\alpha}\frac{\partial}{\partial\alpha}\left(\sin\alpha\frac{\partial}{\partial\alpha}\right) + \frac{1}{q^2\sin^2\alpha}\frac{\partial^2}{\partial\gamma^2}\right] \\ &-\frac{\hbar\omega}{6q^2}\left[\frac{1}{\sin\theta}\frac{\partial}{\partial\theta}\left(\sin\theta\frac{\partial}{\partial\theta}\right) + \frac{1}{\sin^2\theta}\frac{\partial^2}{\partial\phi^2}\right], \end{aligned} \tag{9}$$

and for $n = 2, 4$,

$$\begin{aligned} \tilde{H}_{\mathrm{KE}} = &-\frac{\hbar\omega}{2}\left[\frac{1}{q^2}\frac{\partial}{\partial q}\left(q^2\frac{\partial}{\partial q}\right) + \frac{1}{q^2 \sin\alpha}\frac{\partial}{\partial\alpha}\left(\sin\alpha\frac{\partial}{\partial\alpha}\right) + \frac{1}{q^2\sin^2\alpha}\frac{\partial^2}{\partial\gamma^2}\right] + \frac{\hbar\omega}{3q^2} \\ &-\frac{\hbar\omega}{6q^2}\left[\frac{1}{\sin\theta}\frac{\partial}{\partial\theta}\left(\sin\theta\frac{\partial}{\partial\theta}\right) + \frac{1}{\sin^2\theta}\frac{\partial^2}{\partial\phi^2}\right]. \end{aligned} \tag{10}$$

Here, the coordinates q, α, γ are the radial coordinates and θ, ϕ are the rotational coordinates. Therefore, in the strong coupling limit, the ground state is described by the product of the radial and rotational wave functions,

$$\Psi_0 = \Phi_0^{\mathrm{el}}(\alpha, \gamma, \theta, \phi)\phi^{\mathrm{vib}}(q, \alpha, \gamma)\phi^{\mathrm{rot}}(\theta, \phi), \tag{11}$$

and the ground eigen energy of $\tilde{H}_{\mathrm{JT}}$ is

$$E_0 = -E_{\mathrm{JT}} + \frac{3}{2}\hbar\omega. \tag{12}$$

The first term of the right hand side is the stabilization by the static JT distortion, whereas the right hand side includes the effect of the JT dynamics. Compared with the ground energy of the five-dimensional Harmonic oscillator, $5\hbar\omega/2$, the JT dynamics stabilizes the system by $\hbar\omega$ because of the two rotational modes in the minima of the adiabatic potential energy surface.

On the other hand, when $n = 3$, there are two dimensional radial part and three dimensional rotational part (Eq. (32) in Ref. 1):

$$\tilde{H}_{\mathrm{KE}} = -\frac{\hbar\omega}{2}\left[\frac{1}{q}\frac{\partial}{\partial q}\left(q\frac{\partial}{\partial q}\right) + \frac{1}{q^2}\frac{\partial^2}{\partial\alpha^2} + \frac{9}{4q^2}\right] - \frac{\hbar\omega}{8q^2}\left[4\lambda_x^2 + 4\lambda_y^2 + \lambda_z^2\right], \tag{13}$$

where $\lambda_x, \lambda_y, \lambda_z$ are the angular momenta described by angles γ, θ, ϕ.[1] In the ground state, the eigenstate of H_{JT} (vibronic state) is written as the product of the radial vibration around the minima and the

$$\Psi_0 = \Phi_0^{\mathrm{el}}(\alpha, \gamma, \theta, \phi)\phi^{\mathrm{vib}}(q, \alpha)\phi^{\mathrm{rot}}(\gamma, \theta, \phi), \tag{14}$$

and the ground eigen energy of $\tilde{H}_{\mathrm{JT}}$ is

$$E_0 = -E_{\mathrm{JT}} + \hbar\omega. \tag{15}$$

Compared with the zero vibrational energy of the five-dimensional Harmonic oscillator, there is gain by $3\hbar\omega/2$ due to the JT dynamics. In Ref. 3, the gain by the JT dynamics $3\hbar\bar{\omega}/2$ is evaluated ca 90 meV for C_{60}^{3-}. On the other hand, the frequency for the effective mode is 87.7 meV, and the dynamical component of the ground energy $3\hbar\omega/2 = 132$ meV. The discrepancy is due to the intermediate strength of the orbital vibronic coupling constant g of C_{60}^{n-} anion. In the main text, we assume that, however, the relative strength of the dynamical JT stabilization is the same, and estimate the gain of C_{60}^{4-}.

SUPPLEMENTARY NOTE 2

Exact solution for orbitally disproportionated state and the one-particle exciations in A_4C_{60} with non-hybridized LUMO bands

In bct K_4C_{60}, the effect of the interorbital hybridization of LUMO bands is weak. This is directly observed replacing the interorbital transfer parameter with zero. As an example, we replace the one between the x orbital and the y, z orbitals, (t_{xy}, t_{zx}). Supplementary Figure 6**a** shows the hybridized (red) and non-hybridized (blue) band structures. One finds that these two bands are close to each other in almost all **k**-points and except for around the X point (Supplementary Figure 5). By neglecting the hybridization, the split levels of the hybridized band (around 4.6 eV and 4.9 eV) become quasi-degenerate (around 4.7-4.8 eV). Consequently, the total density of states (DOS) for the non-hybridized band is enhanced around the range of 4.7-4.8 eV and is reduced around 4.6 eV and 4.9 eV (Supplementary Figure 6**b**).

This hybridization effect is diminished by the splitting of the band due to the Jahn-Teller effect and Coulomb repulsion (Supplementary Figure 7). Here, we consider the Jahn-Teller distortion which would give the largest gain of the energy, i.e., x is unstabilized and y and z are stabilized, and $U_\parallel = 0.5$ eV. Therefore, bct K_4C_{60} can be treated as a non-hybridized band system in a good approximation.

We show the band state and electron- and hole-quasiparticle states of non-hybridized multiband Hubbard Hamiltonian:

$$\begin{aligned}\hat{H} = &\sum_{\lambda\mathbf{k}\sigma} \epsilon_{\mathbf{k}\lambda} \hat{n}_{\mathbf{k}\lambda\sigma} + \sum_{\mathbf{m}} \Bigg[\sum_{\lambda} U_\parallel \hat{n}_{\mathbf{m}\lambda\uparrow} \hat{n}_{\mathbf{m}\lambda\downarrow} + (U_\perp - J) \left(\hat{n}_{\mathbf{m}1\uparrow} \hat{n}_{\mathbf{m}2\uparrow} + \hat{n}_{\mathbf{m}1\downarrow} \hat{n}_{\mathbf{m}2\downarrow} \right) \\ &+ U_\perp \left(\hat{n}_{\mathbf{m}1\uparrow} \hat{n}_{\mathbf{m}2\downarrow} + \hat{n}_{\mathbf{m}1\downarrow} \hat{n}_{\mathbf{m}2\uparrow} \right) \\ &+ \sum_{\lambda\neq\lambda'} J \left(\hat{c}^\dagger_{\mathbf{m}\lambda\uparrow} \hat{c}^\dagger_{\mathbf{m}\lambda\downarrow} \hat{c}_{\mathbf{m}\lambda'\downarrow} \hat{c}_{\mathbf{m}\lambda'\uparrow} + \hat{c}^\dagger_{\mathbf{m}\lambda\uparrow} \hat{c}^\dagger_{\mathbf{m}\lambda'\downarrow} \hat{c}_{\mathbf{m}\lambda\downarrow} \hat{c}_{\mathbf{m}\lambda'\uparrow} \right) \Bigg], \end{aligned} \tag{16}$$

where, $\epsilon_{\mathbf{k}\lambda}$ is the band energy.

For simplicity, we first consider that each site has two orbitals $\lambda = 1, 2$, then consider the case with three orbitals $\lambda = 1, 2, 3$.

We assume that the ground state of the system is band insulator type, and orbitals $\lambda = 1$ are doubly occupied and orbitals $\lambda = 2$ are empty for all **k**. The ground state of the

Hamiltonian is given by

$$|\Phi_0\rangle = \prod_{\mathbf{k}\sigma} \hat{a}^\dagger_{\mathbf{k}1\sigma}|0\rangle \tag{17}$$

$$= \prod_{\mathbf{m}\sigma} \hat{c}^\dagger_{\mathbf{m}1\sigma}|0\rangle, \tag{18}$$

where, n is the number of electrons in the system. The ground energy E_0 is directly calculated:

$$\hat{H}|\Phi_0\rangle = \left(\sum_{\mathbf{k}} 2\epsilon_{\mathbf{k}1} + \sum_{\mathbf{m}} U_\parallel\right)|\Phi_0\rangle, \tag{19}$$

therefore,

$$E_0 = \sum_{\mathbf{k}} 2\epsilon_{\mathbf{k}1} + NU_\parallel. \tag{20}$$

Now, we add one electron to empty band orbital $2\mathbf{k}$:

$$|\Phi^e_{\mathbf{k}2\sigma}\rangle = \hat{a}^\dagger_{\mathbf{k}2\sigma}|\Phi_0\rangle, \tag{21}$$

This state is also an eigenstate of $\hat{H}$ (16):

$$\hat{H}|\Phi^e_{\mathbf{k}2\sigma}\rangle = E_{2\mathbf{k}}|\Phi^e_{\mathbf{k}2\sigma}\rangle. \tag{22}$$

Moreover, as in the previous case, Eq. (21) is an eigenstate of each term of the Hamiltonian (electron transfer and bielectronic parts). The first part is obtained as

$$\begin{aligned}\hat{H}_{\mathrm{t}}|\Phi^e_{\mathbf{k}2\sigma}\rangle &= \sum_{\mathbf{k}'\lambda'\sigma'} \epsilon_{\mathbf{k}'\lambda'}\hat{n}_{\mathbf{k}'\lambda'\sigma'}\hat{a}^\dagger_{\mathbf{k}2\sigma}|\Phi_0\rangle \\ &= \hat{a}^\dagger_{\mathbf{k}2\sigma}\sum_{\mathbf{k}'\sigma'} \epsilon_{\mathbf{k}'1}\hat{n}_{\mathbf{k}'1\sigma'}|\Phi_0\rangle + \epsilon_{\mathbf{k}2}\hat{n}_{\mathbf{k}2\sigma}\hat{a}^\dagger_{\mathbf{k}2\sigma}|\Phi_0\rangle \\ &= \left(\sum_{\mathbf{k}'} 2\epsilon_{\mathbf{k}'1} + \epsilon_{\mathbf{k}2}\right)|\Phi^e_{\mathbf{k}2\sigma}\rangle, \end{aligned} \tag{23}$$

where $\hat{H}_{\mathrm{t}}$ is the first term of Eq. (16). The second part is calculated as

$$\hat{H}_{\mathrm{bi}}|\Phi^e_{\mathbf{k}2\sigma}\rangle = \hat{a}^\dagger_{\mathbf{k}2\sigma}\hat{H}_{\mathrm{bi}}|\Phi_0\rangle + [\hat{H}_{\mathrm{bi}}, \hat{a}^\dagger_{\mathbf{k}2\sigma}]|\Phi_0\rangle, \tag{24}$$

where $\hat{H}_{\mathrm{bi}}$ is the second term of Eq. (16). The first term is

$$\begin{aligned}\hat{a}^\dagger_{\mathbf{k}2\sigma}\sum_{\mathbf{m}}\sum_{\lambda} U_\parallel \hat{n}_{\mathbf{m}\lambda\uparrow}\hat{n}_{\mathbf{m}\lambda\downarrow}|\Phi_0\rangle &= \hat{a}^\dagger_{\mathbf{k}2\sigma}NU_\parallel|\Phi_0\rangle \\ &= NU_\parallel|\Phi^e_{\mathbf{k}2\sigma}\rangle. \end{aligned} \tag{25}$$

The second term is

$$
\begin{aligned}
[\hat{H}_{\rm bi}, \hat{a}^\dagger_{\mathbf{k}2\sigma}] &= \sum_{\mathbf{m}} \frac{e^{i\mathbf{k}\cdot\mathbf{m}}}{\sqrt{N}} [\hat{H}_{\rm bi}, \hat{c}^\dagger_{\mathbf{m}2\sigma}] \\
&= \sum_{\mathbf{m}} \frac{e^{i\mathbf{k}\cdot\mathbf{m}}}{\sqrt{N}} \Big\{ U_\parallel [\hat{n}_{\mathbf{m}2\uparrow}\hat{n}_{\mathbf{m}2\downarrow}, \hat{c}^\dagger_{\mathbf{m}2\sigma}] + (U_\perp - J) \left(\delta_{\uparrow\sigma}\hat{n}_{\mathbf{m}1\uparrow}\hat{c}^\dagger_{\mathbf{m}2\uparrow} + \delta_{\downarrow\sigma}\hat{n}_{\mathbf{m}1\downarrow}\hat{c}^\dagger_{\mathbf{m}2\downarrow} \right) \\
&+ U_\perp \left(\delta_{\downarrow\sigma}\hat{n}_{\mathbf{m}1\uparrow}\hat{c}^\dagger_{\mathbf{m}2\downarrow} + \delta_{\uparrow\sigma}\hat{n}_{\mathbf{m}1\downarrow}\hat{c}^\dagger_{\mathbf{m}2\uparrow} \right) \\
&+ J \left(\hat{c}^\dagger_{\mathbf{m}1\uparrow}\hat{c}^\dagger_{\mathbf{m}1\downarrow} (\delta_{\sigma\downarrow}\hat{c}_{\mathbf{m}2\uparrow} - \delta_{\sigma\uparrow}\hat{c}_{\mathbf{m}2\downarrow}) - \delta_{\sigma\uparrow}\hat{c}^\dagger_{\mathbf{m}2\downarrow}\hat{c}^\dagger_{\mathbf{m}1\uparrow}\hat{c}_{\mathbf{m}1\downarrow} - \delta_{\sigma\downarrow}\hat{c}^\dagger_{\mathbf{m}2\uparrow}\hat{c}^\dagger_{\mathbf{m}1\downarrow}\hat{c}_{\mathbf{m}1\uparrow} \right) \Big] ,
\end{aligned}
\tag{26}
$$

and thus,

$$
\begin{aligned}
[\hat{H}_{\rm bi}, \hat{a}^\dagger_{\mathbf{k}2\sigma}]|\Phi_0\rangle &= \sum_{\mathbf{m}} \frac{e^{i\mathbf{k}\cdot\mathbf{m}}}{\sqrt{N}} (2U_\perp - J)\hat{c}_{\mathbf{m}2\sigma}|\Phi_0\rangle \\
&= (2U_\perp - J)|\Phi^e_{\mathbf{k}2\sigma}\rangle,
\end{aligned}
\tag{27}
$$

where $\hat{H}_{\rm bi}$ is the second term of Eq. (16). Therefore, Eq. (21) is an eigenstate of the Hamiltonian (16) and the eigen energy $E^e_{\mathbf{k}2}$ is

$$
E^e_{\mathbf{k}2} = E_0 + \epsilon_{\mathbf{k}2} + 2U_\perp - J. \tag{28}
$$

Similar situation arises when one hole is added. The state is written as

$$
|\Phi^h_{\mathbf{k}1\sigma}\rangle = \hat{a}_{\mathbf{k}1\sigma}|\Phi_0\rangle \tag{29}
$$

and the eigenvalue of the Hamiltonian (16) is

$$
E^h_{\mathbf{k}1} = E_0 - \epsilon_{1\mathbf{k}} - U_\parallel. \tag{30}
$$

From the energies with one electron (28) and one hole (30), the quasi-particle band gap ΔE is obtained as:

$$
\Delta E = \epsilon_{\mathbf{k}2} - \epsilon_{\mathbf{k}'1} + U_\perp - 3J. \tag{31}
$$

Here, $\mathbf{k}$ and $\mathbf{k}'$ are the $\mathbf{k}$-points where the energies of the empty and the filled bands are the minimum and maximum, respectively.

Generalization of the above discussion to the cases with three or more orbitals is straight forward. In the case of three orbitals, we assume that orbitals $\lambda = 1, 2$ are doubly occupied

and orbital $\lambda = 3$ is empty. The form of the eigenstates are the same as before. The eigenstate are given in the main text. The energies are

$$E_0 = \sum_{\mathbf{k}} 2(\epsilon_{\mathbf{k}1} + \epsilon_{\mathbf{k}2}) + N(2U_{\parallel} + 4U_{\perp} - 2J). \tag{32}$$

$$E^e_{\mathbf{k}3} = E_0 + \epsilon_{\mathbf{k}3} + (4U_{\perp} - 2J), \tag{33}$$

$$E^h_{\mathbf{k}\lambda} = E_0 - \epsilon_{\mathbf{k}\lambda} - (U_{\parallel} + 2U_{\perp} - J). \tag{34}$$

Therefore, the energy gap is

$$\Delta E = \epsilon_{\mathbf{k}3} - \epsilon_{\mathbf{k}'\lambda} + U_{\perp} - 3J. \tag{35}$$

Here, $\mathbf{k}$ and $\mathbf{k}'\lambda$ are taken so that the energies of the empty and the filled bands become the minimum and maximum, respectively. These formula hold for the systems with hybridization within the occupied (or empty) bands with slight change.

SUPPLEMENTARY METHODS

Self-consistent Gutzwiller's approach

We briefly explain the Gutzwiller's approach to static Jahn-Teller system developed in Ref. 4. The Gutzwiller's wave function Φ_{G} is written as

$$|\Phi_{\mathrm{G}}\rangle = \hat{P}_{\mathrm{G}}|\Phi_{\mathrm{S}}\rangle, \tag{36}$$

where, Φ_{S} is a Slater determinant,

$$|\Phi_{\mathrm{S}}\rangle = \prod_{\alpha\mathbf{k}\sigma}^{\text{occ.}} \hat{a}^{\dagger}_{\alpha\mathbf{k}\sigma}|0\rangle, \tag{37}$$

and $\hat{P}_{\mathrm{G}}$ is Gutzwiller's projector,

$$\hat{P}_{\mathrm{G}} = \exp\left[-\frac{1}{2}\sum_{\mathbf{m}}\sum_{\lambda\sigma\neq\lambda'\sigma'} A_{\lambda\lambda'}\hat{n}_{\lambda\mathbf{m}\sigma}\hat{n}_{\lambda'\mathbf{m}\sigma'}\right]. \tag{38}$$

Here, we assume the translational symmetry of the system, and the state $\alpha\mathbf{k}$ is a linear combination of the localized states $\lambda\mathbf{m}$:

$$\hat{a}^{\dagger}_{\alpha\mathbf{k}\sigma} = \sum_{\mathbf{m}} \frac{e^{i\mathbf{k}\cdot\mathbf{m}}}{\sqrt{N}} u_{\lambda,\alpha\mathbf{k}}\hat{c}^{\dagger}_{\lambda\mathbf{m}\sigma}, \tag{39}$$

with orbital coefficients $u_{\lambda,\alpha\mathbf{k}}$. N is the number of sites in the system. In Eq. (38), $A_{\lambda\lambda'}$ are real variational parameters. The Gutzwiller's variational parameter $A_{\lambda\lambda'}$ are orbital specific in order to adequately treat the split orbitals.

With the use of the Gutzwiller's wave function (36), the ground state energy per site E_g was calculated. The latter consists of the band energy $E_{\rm band}$, linear Jahn-Teller energy $U_{\rm JT}$, elastic energy $U_{\rm el}$, and bielectronic energy $E_{\rm bi}$:

$$E_g = E_{\rm band} + E_{\rm bi} + E_{\rm JT}. \tag{40}$$

The band energy is written as

$$E_{\rm band} = \sum_{\lambda\lambda'\sigma} q_{\lambda\lambda'} \tau_{\lambda\lambda'}, \tag{41}$$

where $q_{\lambda\lambda'}$ is Gutzwiller's reduction factor, which has the meaning of quasi-particle weight,[5] and $\tau_{\lambda\lambda'}$ is the λ, λ' element of the uncorrelated band energy. For the calculation of $E_{\rm band}$ we used Gutzwiller's approximation.[6,7] The Jahn-Teller energy for C_{60}^{3-} is

$$E_{\rm JT} = \frac{\hbar\omega}{2} q^2 - \sum_\sigma \frac{\sqrt{3}}{2} \hbar\omega g q (n_x - n_y), \tag{42}$$

and for C_{60}^{4-},

$$E_{\rm JT} = \frac{\hbar\omega}{2} q^2 - \sum_\sigma \hbar\omega g q (-2n_x + n_y + n_z). \tag{43}$$

Here, we assume that the Jahn-Teller distortion (Supplementary Table 2) is common to all of the fullerene sites, and q is the magnitude of the deformation. For the bielectronic energy, see Ref. 4.

The total energy E_g contains two types of the variational parameters: orbital coefficients u (39) and Gutzwiller's parameter A (38). The energy E_g is minimized with respect to both u and A. Variational calculations of the energy are performed separately for u an A. From the variation of the energy with respect to u with fixed A, we obtain Hartree-Fock like equation for each $\mathbf{k}$:

$$\sum_{\lambda'} h^{\mathbf{k}}_{\lambda\lambda'} u_{\lambda',\alpha\mathbf{k}} = \epsilon_{\alpha\mathbf{k}} u_{\lambda,\alpha\mathbf{k}}, \tag{44}$$

where $h^{\mathbf{k}}_{\lambda\lambda'}$ is one-electron Hamiltonian, $\epsilon_{\alpha\mathbf{k}}$ is one-electron eigen energy of the Hamiltonian. On the other hand, for fixed u, we minimize E_g with respect to A:

$$\frac{\partial E_g}{\partial A_{\lambda\lambda'}} = 0. \tag{45}$$

These two equations (44), (45) are solved repeatedly until we obtain the convergence of the energy. During the self-consistent calculation, the populations n_λ are fixed, and the ground state for each set of $\{n_\lambda\}$ was performed. For details of the self-consistent Gutzwiller's approach, see Ref. 4.

DFT calculations

Here, we explain tight binding model of bct K_4C_{60} and tight binding parametrization. K_4C_{60} has body centered tetragonal (bct) structure (Supplementary Figure 4). The primitive lattice vector is

$$\mathbf{a}_1 = \frac{1}{2}(a, -a, c), \qquad \mathbf{a}_2 = \frac{1}{2}(a, a, c), \qquad \mathbf{a}_3 = \frac{1}{2}(-a, -a, c), \tag{46}$$

where, a and c are the lattice constants. In the present work, the lattice constants were taken from the neutron diffraction data measured at 6 K ($a = 11.827$ Å, $c = 10.746$ Å).[8] Using $\mathbf{a}_1, \mathbf{a}_2, \mathbf{a}_3$, the nearest neighbor sites (Supplementary Figure 4) are described as

$$\Delta\mathbf{m} = \pm\mathbf{a}_1, \pm\mathbf{a}_2, \pm(-\mathbf{a}_1 + \mathbf{a}_2 + \mathbf{a}_3), \pm\mathbf{a}_3. \tag{47}$$

The next nearest neighbor sites are

$$\Delta\mathbf{m} = \pm a\mathbf{e}_x, \pm a\mathbf{e}_y, \pm c\mathbf{e}_z, \tag{48}$$

where $\mathbf{e}_x, \mathbf{e}_y, \mathbf{e}_z$ are the unit vectors along the axes x, y, z, respectively.

Because of the lower symmetry of bct lattice than fcc one, the orbital energy levels of each site split into three. Thus, the tight-binding model Hamiltonian for the bct lattice is written as the sum of the orbital energy levels part, the electron transfer part between the nearest-neighbor (nn) sites and that between next nearest neighbor (nnn) sites:

$$\hat{H}_{\mathrm{t}} = \sum_{\mathbf{m}} \sum_{\lambda\sigma} \epsilon_\lambda \hat{n}_{\mathbf{m}\lambda\sigma} + \sum_{\mathbf{m}} \sum_{\sigma} \left(\hat{H}^{\mathrm{nn}}_{\mathbf{m}\sigma} + \hat{H}^{\mathrm{nnn}}_{\mathbf{m}\sigma} \right), \tag{49}$$

where the nearest neighbor term is

$$\hat{H}^{\mathrm{nn}}_{\mathbf{m}\sigma} = \sum_{i=1}^{4} \left(t_{xx}\hat{c}^\dagger_{\mathbf{m}+\Delta\mathbf{m}_i x\sigma}\hat{c}_{\mathbf{m}x\sigma} + t_{yy}\hat{c}^\dagger_{\mathbf{m}+\Delta\mathbf{m}_i y\sigma}\hat{c}_{\mathbf{m}y\sigma} + t_{zz}\hat{c}^\dagger_{\mathbf{m}+\Delta\mathbf{m}_i z\sigma}\hat{c}_{\mathbf{m}z\sigma} \right)$$
$$+ \sum_{(\lambda,\lambda')=(y,x),(x,y)} t_{xy} \left(-\hat{c}^\dagger_{\mathbf{m}+\Delta\mathbf{m}_1\lambda\sigma}\hat{c}_{\mathbf{m}\lambda'\sigma} + \hat{c}^\dagger_{\mathbf{m}+\Delta\mathbf{m}_2\lambda\sigma}\hat{c}_{\mathbf{m}\lambda'\sigma} - \hat{c}^\dagger_{\mathbf{m}+\Delta\mathbf{m}_3\lambda\sigma}\hat{c}_{\mathbf{m}\lambda'\sigma} + \hat{c}^\dagger_{\mathbf{m}+\Delta\mathbf{m}_4\lambda\sigma}\hat{c}_{\mathbf{m}\lambda'\sigma} \right)$$
$$+ \sum_{(\lambda,\lambda')=(y,z),(z,y)} t_{yz} \left(-\hat{c}^\dagger_{\mathbf{m}+\Delta\mathbf{m}_1\lambda\sigma}\hat{c}_{\mathbf{m}\lambda'\sigma} + \hat{c}^\dagger_{\mathbf{m}+\Delta\mathbf{m}_2\lambda\sigma}\hat{c}_{\mathbf{m}\lambda'\sigma} + \hat{c}^\dagger_{\mathbf{m}+\Delta\mathbf{m}_3\lambda\sigma}\hat{c}_{\mathbf{m}\lambda'\sigma} - \hat{c}^\dagger_{\mathbf{m}+\Delta\mathbf{m}_4\lambda\sigma}\hat{c}_{\mathbf{m}\lambda'\sigma} \right)$$
$$+ \sum_{(\lambda,\lambda')=(z,x),(x,z)} t_{zx} \left(\hat{c}^\dagger_{\mathbf{m}+\Delta\mathbf{m}_1\lambda\sigma}\hat{c}_{\mathbf{m}\lambda'\sigma} + \hat{c}^\dagger_{\mathbf{m}+\Delta\mathbf{m}_2\lambda\sigma}\hat{c}_{\mathbf{m}\lambda'\sigma} - \hat{c}^\dagger_{\mathbf{m}+\Delta\mathbf{m}_3\lambda\sigma}\hat{c}_{\mathbf{m}\lambda'\sigma} - \hat{c}^\dagger_{\mathbf{m}+\Delta\mathbf{m}_4\lambda\sigma}\hat{c}_{\mathbf{m}\lambda'\sigma} \right)$$
$$+ \mathrm{H.c.} \tag{50}$$

and the next nearest neighbor term is

$$\hat{H}^{\mathrm{nnn}}_{\mathbf{m}\sigma} = t'^{x}_{xx}\hat{c}^\dagger_{\mathbf{m}+\mathbf{e}_x x\sigma}\hat{c}_{\mathbf{m}x\sigma} - t'^{x}_{yy}\hat{c}^\dagger_{\mathbf{m}+\mathbf{e}_x y\sigma}\hat{c}_{\mathbf{m}y\sigma} - t'^{x}_{zz}\hat{c}^\dagger_{\mathbf{m}+\mathbf{e}_x z\sigma}\hat{c}_{\mathbf{m}z\sigma}$$
$$- t'^{y}_{xx}\hat{c}^\dagger_{\mathbf{m}+\mathbf{e}_y x\sigma}\hat{c}_{\mathbf{m}x\sigma} + t'^{y}_{yy}\hat{c}^\dagger_{\mathbf{m}+\mathbf{e}_y y\sigma}\hat{c}_{\mathbf{m}y\sigma} - t'^{y}_{zz}\hat{c}^\dagger_{\mathbf{m}+\mathbf{e}_y z\sigma}\hat{c}_{\mathbf{m}z\sigma}$$
$$- t'^{z}_{xx}\hat{c}^\dagger_{\mathbf{m}+\mathbf{e}_z x\sigma}\hat{c}_{\mathbf{m}x\sigma} - t'^{z}_{yy}\hat{c}^\dagger_{\mathbf{m}+\mathbf{e}_z y\sigma}\hat{c}_{\mathbf{m}y\sigma} + t'^{z}_{zz}\hat{c}^\dagger_{\mathbf{m}+\mathbf{e}_z z\sigma}\hat{c}_{\mathbf{m}z\sigma}$$
$$+ \mathrm{H.c.} \tag{51}$$

Here, ϵ_λ is the orbital energy level, $t_{\lambda\lambda'}$ and $t'_{\lambda\lambda'}$ are the electron transfer parameters between the nearest neighbors and next nearest neighbors.

We obtained the orbital energy levels ϵ and transfer parameters t from the fitting of the DFT band structure to the tight-binding model Hamiltonian (49), (50), and (51). The DFT calculations are described in the Methods and the band structures are shown in Fig. 4**a** in the main text. For the symmetric points indicated in Fig. 4**a**, see Supplementary Figure 5. Supplementary Table 1 shows the derived parameters. The y orbital energy level is lower than the quasidegenerate x and z orbital levels by about 130 meV. We also note that the transfer parameters to the next nearest neighbor sites t' are comparable to the those of the nearest neighbor t. Particularly, the transfer parameter along the z direction t'^{z}_{xx} is the largest than the others. This is explained by the smaller distance between C_{60} sites than the other directions ($c < a$). Therefore, the electron transfer parameters to the next nearest neighbor is crucial to describe the band structure of the bct A_4C_{60}.

SUPPLEMENTARY REFERENCES

[1]M. C. M. O'Brien, "Vibronic energies in ${C_{60}}^{n-}$ and the Jahn-Teller effect," Phys. Rev. B **53**, 3775–3789 (1996).

[2]A. Auerbach, N. Manini, and E. Tosatti, "Electron-vibron interactions in charged fullerenes. I. Berry phases," Phys. Rev. B **49**, 12998–13007 (1994).

[3]N. Iwahara and L. F. Chibotaru, "Dynamical Jahn-Teller Effect and Antiferromagnetism in Cs_3C_{60}," Phys. Rev. Lett. **111**, 056401 (2013).

[4]N. Iwahara and L. F. Chibotaru, "Dynamical Jahn-Teller instability in metallic fullerides," Phys. Rev. B **91**, 035109 (2015).

[5]M. C. Gutzwiller, "Correlation of Electrons in a Narrow s Band," Phys. Rev. **137**, A1726–A1735 (1965).

[6]M. C. Gutzwiller, "Effect of Correlation on the Ferromagnetism of Transition Metals," Phys. Rev. Lett. **10**, 159–162 (1963).

[7]T. Ogawa, K. Kanda, and T. Matsubara, "Gutzwiller Approximation for Antiferromagnetism in Hubbard Model," Prog. Theor. Phys. **53**, 614–633 (1975).

[8]G. Klupp, K. Kamarás, N. M. Nemes, C. M. Brown, and J. Leão, "Static and dynamic Jahn-Teller effect in the alkali metal fulleride salts A_4C_{60} ($A = \mathrm{K, Rb, Cs}$)," Phys. Rev. B **73**, 085415 (2006).
